# Characterization of long non-coding RNA transcriptome in high-energy diet induced nonalcoholic steatohepatitis minipigs

**DOI:** 10.1038/srep30709

**Published:** 2016-07-28

**Authors:** Jihan Xia, Leilei Xin, Wenjuan Zhu, Li Li, Chenxiao Li, Yanfang Wang, Yulian Mu, Shulin Yang, Kui Li

**Affiliations:** 1 State Key Laboratory of Animal Nutrition, Institute of Animal Sciences, Chinese Academy of Agricultural Sciences, No. 2 Yuanmingyuan West Road, Beijing, 100193, P.R. China

## Abstract

Today, obesity and nonalcoholic steatohepatitis are a worldwide epidemic, although how these syndromes are regulated with respect to lncRNAs remains largely unknown. Our previous studies have revealed important pathological features and molecular characteristics of nonalcoholic steatohepatitis in the minipig model, and in this study, we analyze the features of lncRNAs and their potential target genes. Minipig samples only from liver were analyzed using next-generation deep sequencing. In total, we obtained 585 million raw reads approximately 70.4 Gb of high quality data. After a strict five-step filtering process, 1,179 lncRNAs were identified, including 89 differentially expressed lncRNAs (P < 0.05) in the experiment group relative to the control group. The *cis* and *trans* analysis identified target genes that were enriched for specific GO terms (P < 0.01), including immune processes, chemokine activity, cytokine activity, and G-protein coupled receptor binding, which are closely related to nonalcoholic steatohepatitis. The predicted protein-coding targets of the differentially expressed lncRNAs were further analyzed, such as PPAR, FADS2, DGAT2, ACAA2, CYP2E1, ADH4, and Fos. This study reveals a wealth of candidate lncRNAs involved in NASH and their regulated pathways, which should facilitate further research into the molecular mechanisms of this disorder.

Nonalcoholic steatohepatitis (NASH) is an important process in the development of nonalcoholic fatty liver disease, and it is a key stage in the transition from simple steatosis to more severe liver diseases, such as liver fibrosis, hepatocirrhosis, and carcinoma^1^. Nonalcoholic fatty liver disease (NAFLD) is an increasingly common problem worldwide due to changes in modern diets and lifestyles^2^. Excessive lipid accumulation in the liver causes NAFLD, which is closely linked to insulin resistance, obesity, hyperlipidemia, metabolic syndrome, atherosclerosis, and progression to NASH^3^. The mechanism and progression of NASH is not well understood and there are currently no effective treatments. It is known that oxidative stress and inflammatory cytokines play important roles in the pathogenesis and development of NASH, leading to the transition from a fatty liver to more severe conditions such as NASH, fibrosis, or hepatocarcinoma^4^. Several molecular pathways and factors are involved in the progression of NASH, and the most important inflammatory factors are free fatty acids, tumor necrosis factor-alpha, and adiponectin. NASH is an extremely complex liver disease, and the “two-hit”^5^ and “multiple parallel hits”^6^ hypotheses have been proposed to explain the underlying mechanisms. According to these studies, factors such as lipid metabolism, oxidative stress, mitochondrial injury, pro-inflammatory cytokines, gut and adipose derived inflammatory factors, and lipotoxicity all play significant roles in NASH^7^. Furthermore, recent studies have identified additional important factors, such as iron overload, alcohol from the intestines, and high levels of ketone bodies^8^. However, the regulation of NASH-related genes has been less well studied, and potential regulation through long non-coding RNAs (lncRNAs) is now being considered.

It is estimated that as much as 98% of the human genome is transcribed into non-coding RNAs, which were long thought to be “transcriptional noise,” because they had no obvious molecular function and could not be associated with any specific biological processes^9^. However, since then, non-coding RNAs have been shown to play important biological roles in a variety of contexts^10^. Many long non-coding RNAs (lncRNAs) are transcribed throughout the genome and are important for numerous biological pathways and the development of human disease^11^,^12^. LncRNAs are defined as RNA molecules greater than 200 nucleotides in length that lack an open reading frame (ORF), and they can be subdivided into long intergenic ncRNAs (lincRNAs), long intronic ncRNAs, antisense RNAs, pseudogene RNAs, and other types of RNA molecules^9^. LncRNAs often have low expression levels, and they can display tissue specificity and evolutionary conservation^13^. LncRNAs have been shown to play roles in chromatin modification^14^, the pairing of homologous or non-homologous chromosomes, the pluripotency of embryonic stem cells^15^, development, and epigenetic regulation. The regulatory modes of lncRNAs can involve *cis* interactions to affect the expression of nearby genes or protein complexes or *trans* interactions to regulate more distantly located genes^16^, interacting chromatin complexes, or transcription factors. Furthermore, lncRNAs can interact with DNA, RNA, and proteins to both activate and suppress gene expression, and they can bind to transcription factors or chromosome-regulating complexes^17^. As important regulatory molecules, lncRNAs are likely to play an important role in NASH development.

In our previous studies on early nonalcoholic steatohepatitis, in which we induced this disease using a high-fat, high-sucrose diet for 23 months in Bama minipigs, we showed that an important mechanism of NASH was a hyperinsulinemia-based shift in the energy supply from glucose to ketone bodies^18^. Pigs with early NASH displayed obesity, metabolic syndrome, hyperlipidemia, and hyperinsulinemia, and the liver pathology characteristics revealed microvesicular steatosis, inflammation^19^, lipid peroxidation, iron overload, decreased antioxidant capacity, and cellular damage. The gene expression levels were characterized by *de novo* fatty acid synthesis deposited as lipid droplets in liver cells, and the high ketone levels caused cytochrome P450 2E1 overexpression, iron overload, and CYP2E1 and alcohol dehydrogenase 4 overexpression, leading to reactive oxygen species (ROS) production, which ultimately caused DNA and cellular damage. To perform a more in-depth study of the mechanisms of NASH at the level of RNA library samples from the livers of early NASH minipigs were sequenced using next-generation deep sequencing technologies.

## Results

### The characteristics of Bama minipigs fed a high-fat, high-sucrose diet for 23 months

The body weight of the HFHSD pigs was significantly higher than that of the control pigs due to the higher amount of visceral and subcutaneous fat. Serum indices, such as insulin, triglycerides, and low-density lipoprotein cholesterol, were significantly higher in the HFHSD group than in the control group. The livers of the HFHSD pigs exhibited large numbers of inflammatory cells, including lymphocytes, eosinophils, neutrophils, and Kupffer cells. By contrast, serum glutathione peroxidase and total antioxidant capacity decreased, indicating that the livers were undergoing oxidative stress and inflammatory responses^18^. Because lncRNAs play important roles in many biological processes, we used deep sequencing analysis to determine how lncRNAs affect NASH development following induction with a high-energy. Several important lncRNAs (Table S1) regulated target genes which are closely related pig NASH model, were analyzed in this study.

### Reads and mapping results of RNA deep sequencing

**Figure 1:**
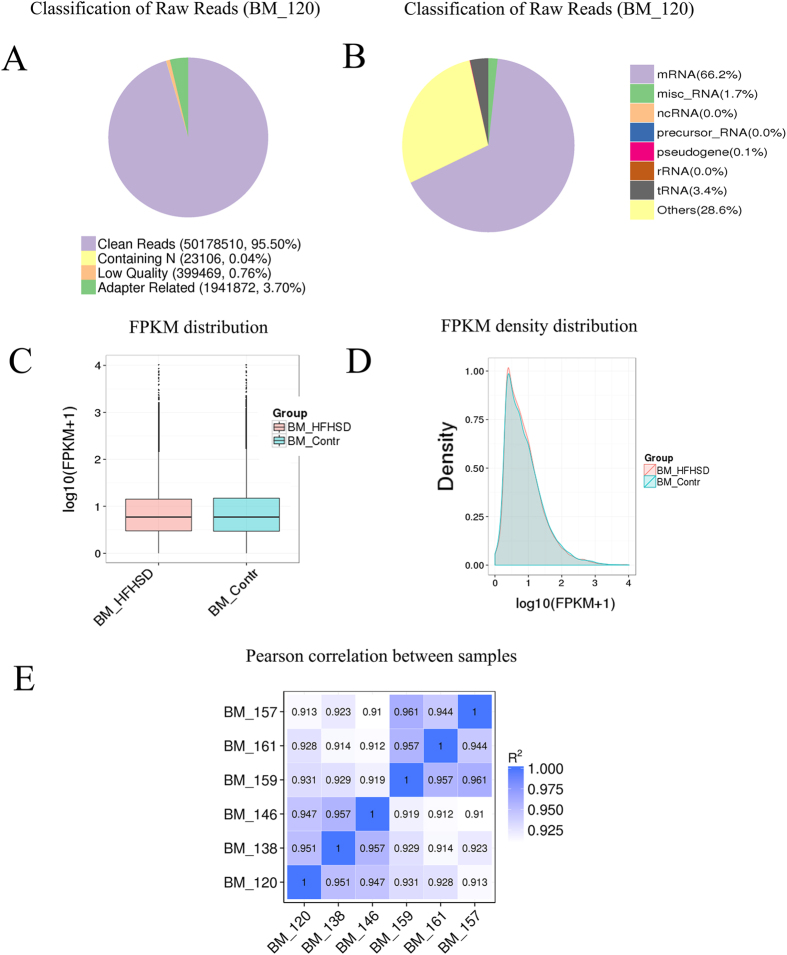
Reads and mapping results of RNA deep sequencing. (**A)** The quality of the raw reads. **(B)** Classification of raw reads, mRNA near 60%, followed by tRNAs, microRNAs, and pseudogenes for pig 120; other information is shown in Supplementary Figure 2. **(C)** The FPKM distribution is shown with a box plot. **(D)** FPKM density distribution for all transcripts. **(E)** The Pearson correlation coefficients for all pigs.

The sequencing analyses were performed by the Novogene Bioinformatics Technology Company. Three pigs in the HFHSD group and three pigs in the control group were included in this study. In total, 585 million raw reads were acquired with an error rate less than 0.04%. After filtering the raw reads and removing low-quality reads, there were approximately 563 million clean reads, yielding approximately 70.4 Gb of high-quality data and covering 95% of the total genome (Table S2). Pig 120 for example had 95.5% clean reads (Fig. 1A). The clean reads were mapped to the pig genome v10.2, and the total mapped reads were more than 80%. The uniquely mapped reads were greater than 60%, non-splice reads were more than 39%, and splice reads were approximately 18% (Table S3). The majority of reads were mRNAs (~60%), followed by tRNAs, miscRNAs, and others (Fig. 1B, Table S4).

#### Assessing the overall quality of the RNA-seq data

In this study, the expected FPKM (Fragments Per Kilobase of transcript per Million mapped reads) values were used to calculate gene expression levels. The distribution profile for all transcripts is shown as a box plot in Fig. 1C, and the FPKM density distribution is shown in Fig. 1D. The patterns of expression between the HFSHD group and control group were similar, with only slight differences. The vertical scale of Fig. 1C represents log_10_ (FPKM+1) values in the HFHSD and control group. Figure 1D shows that the maximum density of log_10_ (FPKM+1) was approximately 0.5, and the FPKM increased as the density gradually decreased. The RNA-Seq Pearson correlation coefficients of gene expression levels were greater than 0.95 in the HFHSD group and greater than 0.94 in the control group, demonstrating the rationality of the experimental design between the two groups and the similarity of expression within the groups (Fig. 1E).

**Figure 2:**
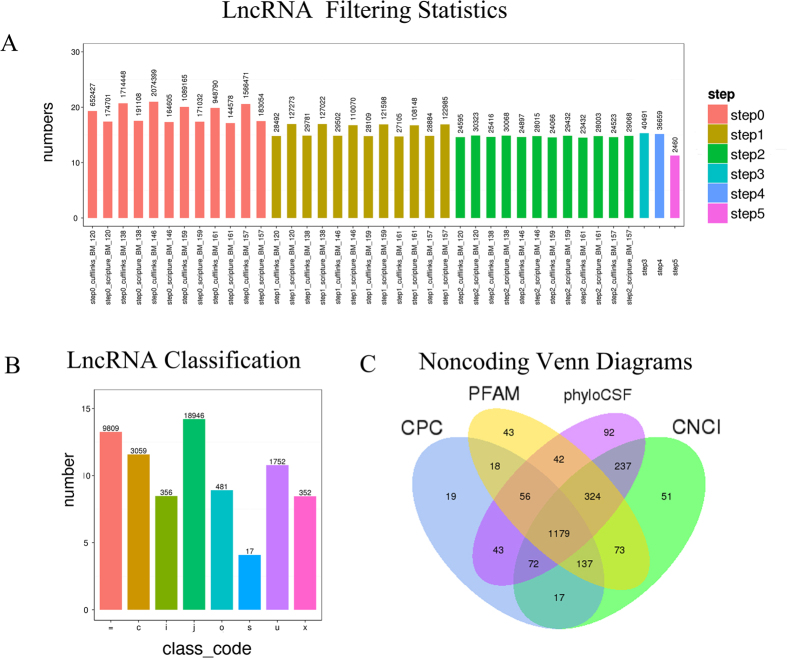
Filtering statistics, classification, and coding potential screening of the lncRNAs. (**A)** Statistics for the number of transcripts remaining during the filtering process (step 1 to 5). **(B)** The classifications are shown as eight types. = for complete match of intron chain; c for contained; j for potentially novel isoform; i for a transfrag falling entirely within a reference intron; o for generic exonic overlap with a reference transcript; u for unknown, intergenic transcript; x for exonic overlap with reference on the opposite strand; s for intron of the transfrag overlaps a reference intron on the opposite strand. **(C)** Coding potential was analyzed using four methods.

Two methods, Cufflinks^20^ and Scripture^21^, were used to splice the lncRNAs. After five steps filtered, 2,460 lncRNAs were selected, including 1,752 lincRNAs, 356 intronic lncRNAs, and 352 anti-sense lncRNAs (Fig. 2B). The key criteria for whether a transcript was deemed a lncRNA was whether it had coding potential. The final set of lncRNAs was the intersection of the sets from four commonly used analysis methods: CPC, CNCI, pfam Protein Domains, and PhyloCSF. All non-coding transcripts for each method were as follows: 1,541 for CPC, 2,090 for CNCI, 1,872 for pfam, and 2,045 for phyloCSF (Table S5). Non-coding Venn diagrams are shown, and they illustrate the number of common and unique transcripts among the four methods. All of the lncRNA sequences in early NASH minipigs were assembled, and they are reported for the first time in this study (Table S6). The set of 1,179 lncRNAs from the intersection of the four methods was used for subsequent analyses (Fig. 2C).

**Figure 3:**
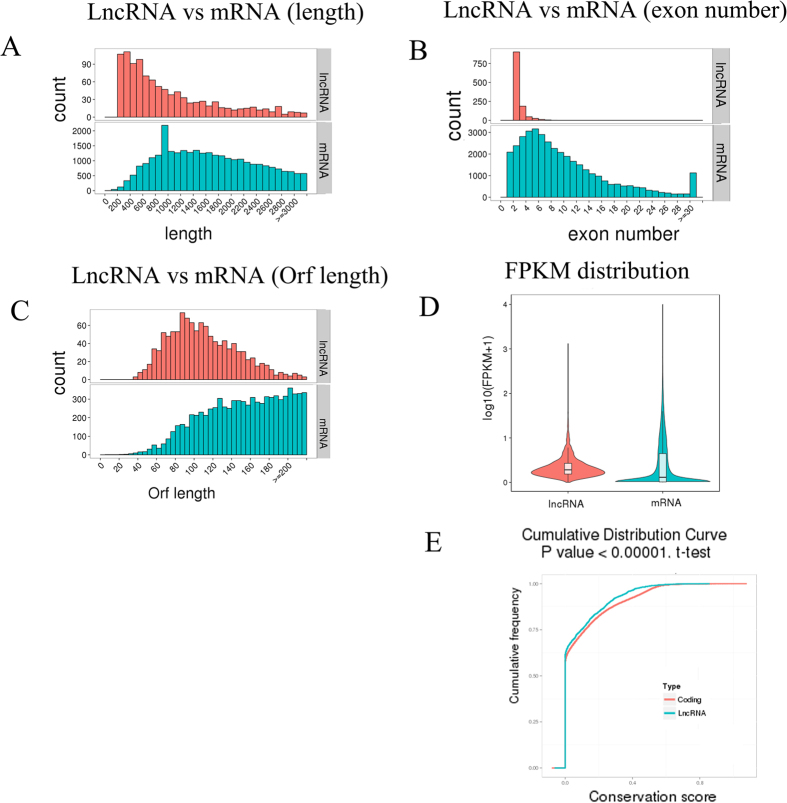
Structural comparison between lncRNAs and mRNAs. The lncRNA and mRNA transcripts compared by length **(A)**, exon number **(B)**, Orf length **(C)**, FPKM **(D, E)**, and conservation **(F)**.

The cuffdiff software was used to analyze the quantitative expression levels of lncRNAs for read counts and FPKM. The lengths of the lncRNAs were between 200 bp and 800 bp, and the average length was approximately 300 bp. The number of transcripts decreased significantly as the lncRNAs became longer; in contrast mRNAs were generally between 800 bp and 2,000 bp (Fig. 3A). Most lncRNAs contained 2 exons, although a small number of transcripts had more than 8 exons. Most of the mRNAs had approximately 6 exons, with fewer transcripts being observed with more exons (Fig. 3B). The ORFs of the mRNAs were acquired using structural annotation with known genes, and the sequences were predicted using Estscan. The ORF sequences were translated to protein sequences. The length of the predicted ORFs in the lncRNAs was centered approximately 80, and this number decreased with increasing ORF lengths. In contrast, the lengths of most of the mRNA ORFs were concentrated between 100 and 200 (Fig. 3C). The difference in expression levels between lncRNAs and mRNAs was expressed as the log_10_ (FPKM+1) value of the average of the two groups. The average lncRNA expression level was higher than the mRNA average expression level. The differences between lncRNAs and mRNAs are shown in Fig. 3D. PhastCons was used to analyze the conservation of mRNAs and lncRNAs, and the coding RNAs were more highly conserved than the lncRNAs (Fig. 3E).

**Figure 4:**
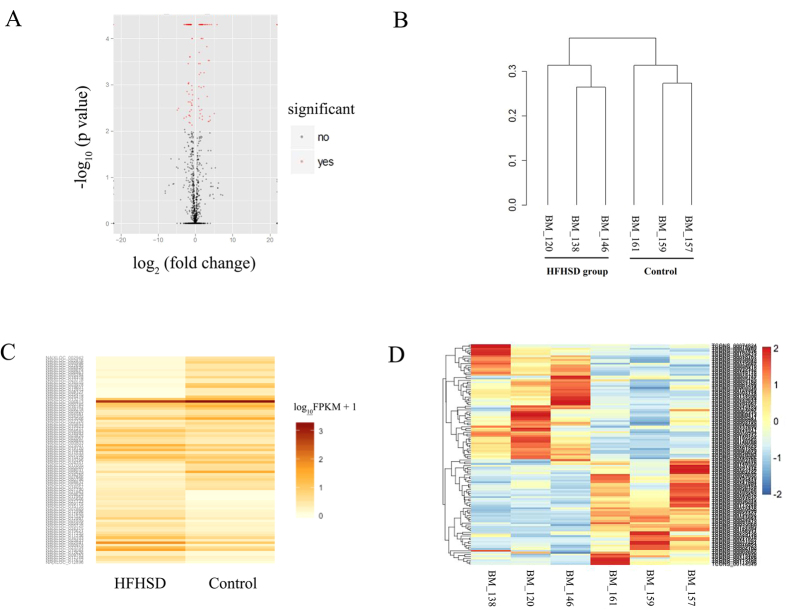
Characteristics of lncRNA expression levels between the HFHSD and control groups. All lncRNA expression levels are shown; differentially expressed lncRNAs are shown in red and the others in black (**A**). A cluster dendrogram of the lncRNAs analyzed in six minipigs (**B**). The Heat map shows the expressed lncRNAs (P < 0.05) in the two groups (**C**). The differentially expressed lncRNAs in three pigs in the HFHSD group and three pigs in the control group were analyzed for cluster patterns (**D**).

The selected lncRNAs were quantified using cuffdiff software for the read count and RPKM analyses. The graphs in Fig. 4A show the lncRNA expression levels, and the differentially expressed lncRNAs are shown in red (Fig. 4A). There were 89 differentially expressed lncRNAs (P < 0.05). In particular, TCONS_00020037 was down-regulated 34-fold, and TCONS_00132537 was up-regulated 29-fold (Table S7). A cluster dendrogram of the lncRNAs was constructed using the data from the minipigs of both the HFHSD group and control group. The graph in Fig. 4B shows the consistency between the two groups (Fig. 4B). The Heat map (Fig. 4C) shows the differentially expressed lncRNAs (P < 0.05) between the HFHSD group and the control group. The differentially expressed lncRNAs in the pigs from the HFHSD and control groups were analyzed for cluster patterns. The expression patterns were clearly different between the HFHSD group and control group, and the expression patterns were consistent (Fig. 4D). We selected eight lncRNAs such as XLOC_009593, XLOC_001424, XLOC_008628, XLOC_019865, XLOC_017148, XLOC_017612, involved in the regulation of lipid metabolism, chemokines, and immune responses for validation using qRT- PCR in *trans*, and the results were found consistent with differential expression observed in RNA-seq analysis (Figure S1). These lncRNAs regulated target gene such as PPAR, FADS2, DGAT2, ACAA2, CYP2E1, ADH4, and Fos. XLOC_009593 and its target gene PPAR were downregulated. Other lncRNAs and their target genes were upregulated.

All reads were assembled into 39,398 transcripts as either mRNAs or lncRNAs. In total, there were 38,219 mRNAs, including 1,725 differentially expressed mRNAs (P < 0.05). Of these 1,725 mRNAs, 964 mRNAs were altered by >2-fold change in expression (Table S7); and 1,179 lncRNAs, with 89 lncRNAs identified as being differentially expressed (P < 0.05). Of these 89 lncRNAs, 78 differed in expression by >2-fold; elsewhere where 2-fold is written, the change was >2-fold (Table S8). The differentially expressed mRNAs were analyzed for GO enrichment, as shown in the supplemental material (Figure S2, Table S7). GO analysis showed that terms were expressed differentially including catalytic activity, oxidoreductase activity, the metabolic process, the oxidation-reduction process, and the immune response. These terms are closely related to liver metabolic syndrome in NASH. We analyzed the differentially expressed mRNAs for KEGG enrichment and the pathways are shown in Table S7. The differentially expressed pathways are metabolic pathways, retinol metabolism, alanine, aspartate and glutamate metabolism; the biosynthesis of amino acids; the citrate cycle (TCA cycle); and antigen processing and presentation.

The number of lncRNAs involved in the GO enrichment and KEGG pathways was lower than the number of differentially expressed mRNAs. LncRNAs were mainly regulated in several important pathways such as chemokine receptor binding, metabolic pathways in *cis*, carbon metabolism, oxidative phosphorylation, and the immune response in *trans*. The differentially expressed mRNAs covered nearly all the GO terms and pathways of lncRNAs regulated in metabolic pathways and immune pathways. Furthermore, the most important differentially expressed genes, including those involved in glucose and lipids metabolism and the immune response, are regulated by lncRNAs. The differentially expressed GO terms and pathways illustrated that lncRNAs play an important role in the development of NASH especially in metabolic and immune responses.

## Discussion

LncRNAs play important roles in the regulation of gene expression, transcription, and post-transcriptional modification and the lncRNAs found in these patients are similar to those found in our other experiments^22^. LncRNAs could represent a new approach for designing therapeutic and diagnostic methods, and new drugs might be screened by the direct detection of these RNAs^23^. However, the mechanisms of lncRNAs affecting NASH are not yet understood. In recent years, minipigs have become a widely used experimental animal model in human disease research, particularly for treatment strategies and the development of novel drugs due to their similarities with humans in terms of energy metabolism, cardiovascular systems, lack of brown fat, and organ size^24^. The high-fat, high-energy diet used in this study produced obesity, fatty livers, cardiac hypertrophy, and fatty hearts in the pigs^25^. In this study, various differentially expressed lncRNAs were identified that appear to regulate target protein-coding genes that are closely associated with NASH, such as PPAR alpha, HMGCS2, FADS2, DGAT2, ACAA2, CYP2E1, ADH4, and Fos. The lncRNAs (Table S9) are predicted as the regulator for these genes such as XLOC_009593, XLOC_001424, XLOC_008628, XLOC_019865, XLOC_017148, XLOC_017612 etc. Every target gene may be regulated by several lncRNAs in *cis* or *trans*, so further researches need to perform to study these mechanism. PPAR alpha as target gene of XLOC_009593 has been shown in NASH animal models to be associated with many inflammatory agonist-dependent mechanisms that affect other transcription factors, such as AP1, STATs, and NFAT^26^. As a transcriptional activator of HMGCS2^27^, peroxisome proliferator-activated receptor alpha, which is target gene of XLOC_001424 and both upregulated, affects the energy balance between glucose and ketone bodies within the mitochondria^18^. Indeed, energy metabolism disorders are an important aspect of NASH, as indicated by the fact that insulin resistance promotes the production of arachidonic acid (FADS2)^28^ and TG (DGAT2)^29^ and affects mitochondrial fatty acid beta-oxidation by acetyl-CoA acyltransferase 2 (ACAA2)^30^, which is the target of XLOC_023704 in early NASH minipigs, and the target gene and lncRNA both upregulated. High levels of ketone bodies are caused by CYP2E1^31^ which is the target gene of XLOC_017612, and ADH4 overexpression, which leads to the production of ROS^18^. This study identified many lncRNAs that may be involved in early NASH in minipigs. However, future studies will be needed to validate these predictions and determine the role of these lncRNAs in NASH.

**Figure 5:**
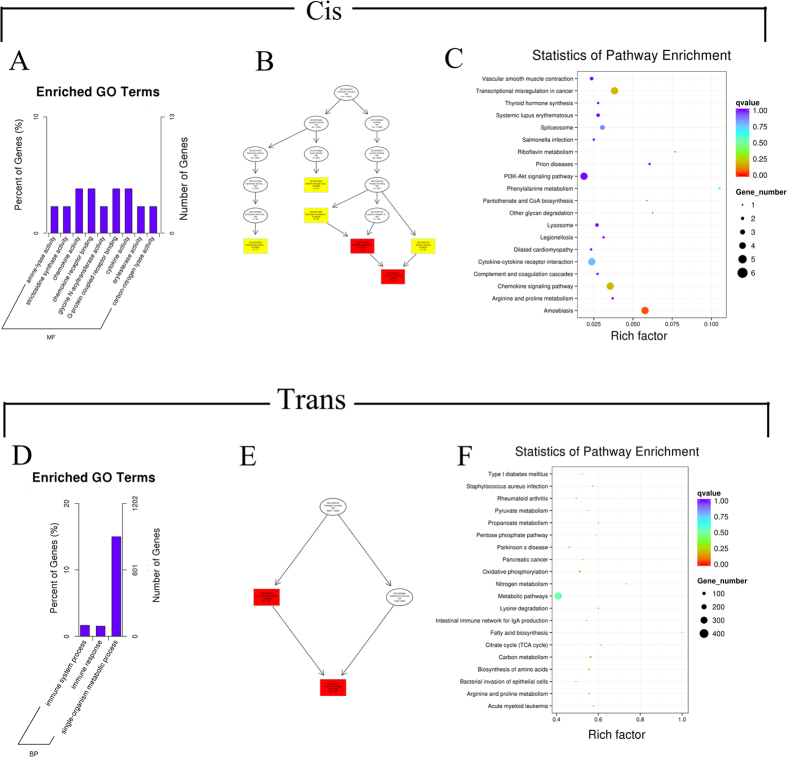
The prediction and functional analysis of target genes regulated by differentially expressed lncRNAs between the HFHSD and control groups. For *cis* interactions, lncRNA target gene GO enrichment histogram (**A**), lncRNA target gene GO terms DAG figure, MF means molecular function, BP means biological process (**B**), and target genes KEGG enrichment scatter plot (**C**). For *trans* interactions, GO enrichment histogram (**D**), GO terms DAG (**E**), and KEGG enrichment scatter plot (**F**).

LncRNAs can regulate target genes in *cis* or in *trans*, both of which could be important for the pathology and biological processes of NASH. A functional enrichment analysis of differentially expressed lncRNA target genes was carried out to search for relevant GO terms and pathways by *cis* and *trans* in early NASH minipigs (Fig. 5). The rationale for identifying *cis* target genes was that the lncRNAs should be in relatively close proximity to the protein-coding genes. Therefore, all genes in the proximity of the lncRNA loci (10 kb or 100 kb upstream or downstream) were selected as target genes, and the enrichment of specific molecular functions among the target genes was analyzed to predict the functions of the lncRNAs. There were 248 genes within 10 kb of the lncRNAs, and 2,048 genes within 100 kb (Table S9). The distribution of the target genes according to GO revealed differences in terms of gene function. GO analysis showed that 9 GO terms were expressed differentially (Table S10), including chemokine activity, chemokine receptor binding, cytokine activity, and G-protein coupled receptor binding, which are closely related to liver metabolic syndrome in NASH. Various chemokines regulate the inflammatory response and interact with different types of liver cells, such as Kupffer cells, stellate cells, endothelial cells, and immune cells^32^. Cytokines, such as tumor necrosis factor, interleukin 8, and transforming growth factor are involved in NASH, both through the inflammatory response and superoxide damage^33^.

The target genes of the lncRNAs in *cis* that were enriched for specific GO and KEGG pathways are shown in Table S10. Directed Acyclic Graph (DAG) was used for the graphical representation of lncRNA target gene GO enrichment analysis. The GO branches represent the contained relationships. From the top to the bottom, the defined functions became smaller. The GO terms shown in yellow represent differences in arylesterase activity, carbon-nitrogen lyase activity, G-protein coupled receptor binding, and cytokine activity, and the GO terms shown in red represent significant differences in chemokine activity and chemokine receptor binding (Fig. 5B). A GO enrichment histogram intuitively shows the number of genes for a term distributed across biological processes, cellular components, and molecular functions. The histogram shows the enriched molecular function GO terms, including chemokine receptor binding, G-protein coupled receptor binding^34^, cytokine activity^35^, and chemokine receptor binding^36^, with detailed information shown in Table S10 (Fig. 5A).

The main biochemical metabolic pathways and signal transduction pathways of the differentially expressed lncRNA-regulated target genes were determined by pathway enrichment. The scatter plot is a graphical representation of the statistical analyses that visualizes the pathway enrichment. The degree of KEGG enrichment was measured in terms of richness factor, P value, and the number of genes in the pathway. The important enriched pathways with low P-values and large numbers of genes are shown in the figure, and they include the PI3K-Akt signaling pathway, vascular smooth muscle contraction, cytokine-cytokine receptor interaction, spliceosome, chemokine signaling pathway, and arginine and proline metabolism. These enriched pathways are primarily related to cytokine signaling and the inflammatory response, and the chemokine signaling pathway is likely related to NASH pathology (Fig. 5C, Table S10) in Bama minipigs in whom NASH was induced by a long-term high-energy diet.

### Differentially expressed lncRNA *trans*-regulated target genes from the GO and KEGG enrichment analyses

The predictions for *trans* target genes were based on the principle that the functions of lncRNAs do not depend on gene location, but can be identified by co-expression with their protein-coding target genes. Therefore target genes were predicted based on correlations between lncRNA and mRNA expression levels^37^. The *trans* analysis results showed that the differentially expressed lncRNAs regulated numerous target genes (Table S11). The *trans*-regulated target gene GO terms identified immune system processes and immune responses as the biological processes (Fig. 5E). The immune system process and immune response GO terms were significant as biological processes among all the target genes (Fig. 5D). The differentially expressed chemokine signaling pathway is closely related to NASH development^36^. The KEGG enrichment analysis showed that the lncRNAs were mainly enriched in the regulation of metabolic pathways. The most highly enriched pathways, such as fatty acid biosynthesis^38^, carbon metabolism, and amino acid metabolism^39^, are closely linked to the mechanisms of early NASH. Other obvious pathways, such as carbon metabolism, oxidative phosphorylation, biosynthesis of amino acids, nitrogen metabolism, citrate cycle (TCA cycle), and fatty acid biosynthesis^40^, probably also play important roles in the development of early NASH (Fig. 5F).

## Conclusions

In conclusion, this study revealed many important molecular characteristics of lncRNAs and their target genes in early minipigs induced with NASH by a long-term high-fat, high-sucrose diet. Our aim was to elucidate regulatory mechanisms of NASH development in minipigs. Using next-generation deep sequencing, this study is the first to provide information concerning lncRNAs in this model. We identified many differentially expressed lncRNAs between the HFHSD group and the control group, and we predicted and analyzed target genes for GO term and KEGG pathway enrichment. This study provides a wealth of information on lncRNAs in minipigs, including statistics, distributions, and differences between expressed genes, as well as GO term and pathway analyses for potential targets. This data could yield novel insights into the molecular mechanisms of NASH and help develop novel diagnostic and therapeutic strategies for this disease.

## Methods

### Minipigs and experimental design

Twelve six-month-old Bama pigs of the 18th generation of either sex were used in this experiment. All pigs were fed in individual pens under controlled conditions (temperature 18 °C–22 °C; relative air humidity 30–70%) and were fed twice daily on a restricted schedule and dietary dose [3% of body weight monthly; facility certification No.: SYXK (Beijing) 2008–007]. The miniature pigs used in this experiment were treated humanely according to the “Guide for the Care and Use of Laboratory Animals, ISA, CAAS”, and all procedures were approved by the Animal Care and Use Committee of the Germplasm Resource Center of Chinese Experimental Minipigs. The pigs were provided free access to water for 23 months. Six pigs were fed a controlled diet and another six pigs were fed a high-fat, high-sucrose diet (HFHSD; 53% control diet, 10% pork lard, and 37% sucrose). At the end of the experiment, pigs were fasted overnight and euthanized with an overdose of ketamine and xylazine.

### RNA isolation, library preparation, and sequencing

RNA isolation, library construction, and sequencing were performed by Novogene Corporation. RNA extraction was performed according to the manufacturer’s instructions and was quantified using a Bioanalyzer 2100 (Agilent Technologies, CA, USA). The quality and quantity of RNA were assessed using an RNA Assay Kit and a fluorometer (Life Technologies, CA, USA). RNA was frozen and stored at −80 °C immediately after production.

A total quantity of 3 μg RNA per sample was used as the input material for the RNA sample preparations from three HFHSD pigs (number as 120, 138, 146) and three control pigs (number as 157, 159, 161). First, ribosomal RNA was removed using an rRNA Removal Kit (Epicentre, WI, USA), and the rRNA-depleted sample was cleaned. Subsequently, sequencing libraries were generated. To select cDNA fragments between 150–200 bp in length, the library fragments were purified with an appropriate system (Beckman Coulter, Beverly, USA). Subsequently, USER enzyme was incubated with the size-selected, adaptor-ligated cDNA samples. PCR was performed using Phusion High-Fidelity DNA polymerase, Universal PCR primers, and an Index Primer. Finally, the products were purified and library quality was assessed. After cluster generation, the libraries were sequenced on an Illumina Hiseq 2000 platform, and 100-bp paired-end reads were generated.

### Quality control

Raw reads in a fastq format were first processed using in-house perl scripts. During this step, clean reads were obtained by removing all reads containing an adapter, reads containing poly-N sequences, and low-quality reads from the raw data. At the same time, Q20, Q30, and GC content were calculated for the “clean” dataset. All downstream analyses were based on the clean data. The raw sequencing data are available from the NCBI and are archived under the accession number SRX1465952.

### Mapping to the reference genome

The reference genome and gene model annotation files were downloaded directly from the genome website. An index of the reference genome was built using Bowtie v2.0.6, and paired-end clean reads were aligned to the reference genome using TopHat v2.0.9.

### Transcriptome assembly

The mapped reads of each sample were assembled using both Scripture (beta2)^21^ and Cufflinks (v2.1.1)^20^ with a reference-based approach. Both methods employed spliced reads to determine exon connectivity, albeit using two different approaches. Scripture uses a statistical segmentation model to distinguish expressed loci from experimental noise and uses spliced reads to assemble the expressed segments, after which it reports all statistically expressed isoforms in a given locus. By contrast, Cufflinks uses a probabilistic model to simultaneously assemble and quantify the expression levels of a minimal set of isoforms, which provides a maximum likelihood explanation of the expression data in a given locus. Scripture was run with default parameters. The lncRNA candidates were filtered using five strict steps based on RNA structural characteristics and non-coding properties. The five steps were as follows: step 1) the length of the transcript must be ≥200 bp, and the number of exons must be ≥2; step 2) Cufflinks was used to calculate the read coverage, and the transcript coverage must be ≥3; step 3) Using the predicted splicing transcripts from Cufflinks and Scripture, the candidate transcripts must have been expressed in at least two samples; step 4) transcripts that were similar to known swine non-coding RNAs and non-mRNAs (rRNAs, tRNAs, snRNAs, snoRNAs, pre-miRNAs, and pseudogenes) were removed; and step 5) after comparisons with known mRNAs, the remaining long non-coding RNAs were separated into lincRNAs, intronic lncRNAs, and anti-sense lncRNAs using the class_code module in cuffcompare.

### Four methods of Coding potential analysis

Coding-Non-Coding-Index (CNCI) profiles of adjoining nucleotide triplets were analyzed to effectively distinguish protein-coding and non-coding sequences independent of previous annotations^41^. The Coding Potential Calculator (CPC) primarily assesses the extent and quality of the ORF in a transcript and searches sequences against a known protein sequence database to discriminate between coding and non-coding transcripts^42^. We translated each transcript in all three possible frames and used Pfam Scan (v1.3) to identify any known protein family domains documented in the Pfam database (release 27; used both Pfam A and Pfam B)^43^ or the NCBI eukaryote protein database. Phylogenetic codon substitution frequency (PhyloCSF) examines alignments of conserved coding regions to detect evolutionary signatures that can be used to distinguish between protein-coding and non-coding transcripts^44^. Transcripts that were predicted to have coding potential by any of the four above tools were removed from the dataset, and the remaining transcripts without coding potential were employed as our candidate set of lncRNAs.

### Conservative analysis

Phast (v1.3) is a software package that contains many statistical programs for use in phylogenetic analyses^45^, and phastCons is a conservation scoring, identification program for conserved elements. We used phyloFit to compute phylogenetic models for conserved and non-conserved regions between species and then built the model and HMM transition parameters in phastCons to compute a set of conservation scores for the lncRNA and coding genes.

### *Cis* and *trans* target gene prediction

The term *cis* refers to a situation in which lncRNAs act on neighboring or nearby target genes. We searched for coding genes within 10 kb to 100 kb upstream or downstream of each lncRNA and then analyzed their functions. For *trans* interactions, we searched for lncRNAs that correlated with gene expression levels. Because there were fewer than 25 samples, we correlated expression levels between lncRNAs and coding genes using custom scripts.

### Quantification of gene expression levels

Cuffdiff (v2.1.1) was used to calculate FPKMs for both lncRNAs and coding genes in each sample^20^. Gene FPKMs were computed by summing the FPKMs of the transcripts in each gene group. FPKM stands for “fragments per kilo-base of exon per million fragments mapped,” and it is calculated based on the length of the fragments and the reads count mapped to each fragment. Transcripts with prefix NM or XM are mRNAs which are from pig reference transcripts database, and the prefix TCONS is for lncRNAs.

### Differential expression analysis

Cuffdiff provides statistical routines for determining differential expression in digital transcript or gene expression datasets using a model based on a negative binomial distribution. For biological replicates, transcripts or genes with P values less than 0.05 were assigned as differentially expressed. For non-biological replicates, P < 0.05 and the absolute value of log2 (fold change) < 1 were set as the threshold for significant differential expression.

### Gene Ontology (GO) and KEGG enrichment analysis

GO enrichment analysis of differentially expressed genes or lncRNA target genes was implemented with the GOseq R package, with gene length bias corrected. GO terms with corrected P values less than 0.05 were considered to be significantly enriched among the differentially expressed genes. We used the KOBAS software program to test for the statistical enrichment of the differentially expressed genes or lncRNA target genes among the KEGG pathways. Statistical correction was implemented for multiple hypothesis testing for GO term and KEGG pathway enrichment analysis.

### Validation of RNA-Seq data by qRT-PCR

qRT-PCR was used to confirm the RNA-Seq results for eight lncRNAs transcripts. The PCR primers designed for the lncRNAs are shown in Table S1. Relative gene expression levels are expressed in threshold cycle (Ct) values. Ct values were averaged across each reaction, and lncRNAs levels were normalized to those of glyceraldehyde-3-phosphate dehydrogenase (GAPDH). Ct values were averaged, and the difference between the averaged GAPDH Ct and the gene of interest averaged Ct was calculated using the 2^−ΔΔCT^ (∆∆CT = (Ct treated − Ct control)). The relative expression levels of the lncRNAs of interest were analyzed using the 2^−ΔΔCT^ method, and the data analyzed were from three different experiments.

## Additional Information

**How to cite this article**: Xia, J. *et al*. Characterization of long non-coding RNA transcriptome in high-energy diet induced nonalcoholic steatohepatitis minipigs. *Sci. Rep.* **6**, 30709; doi: 10.1038/srep30709 (2016).

## Supplementary Material

Supplementary Information

Supplementary Table S5

Supplementary Table S6

Supplementary Table S7

Supplementary Table S8

Supplementary Table S9

Supplementary Table S10

Supplementary Table S11
