# Supplementary material for: Characterization of long non-coding RNA transcriptome in high-energy diet induced nonalcoholic steatohepatitis minipigs
Source: Sci Rep. 2016 Jul 28;6:30709. doi: 10.1038/srep30709 (PMC4964571; doi:10.1038/srep30709)
Supplement: Supplementary Information [file srep30709-s1.docx]

**Characterization of long non-coding RNA transcriptome in high-energy diet induced nonalcoholic steatohepatitis minipigs**

Jihan Xia^1^, Leilei Xin^1^, Wenjuan Zhu, Li Li, Chenxiao Li, Yanfang Wang, Yulian Mu, Shulin Yang^1^ *, Kui Li^1^,*

^1^State Key Laboratory of Animal Nutrition, Institute of Animal Sciences, Chinese Academy of Agricultural Sciences, No.2 Yuanmingyuan West Road, Beijing, 100193, P.R. China

*Corresponding author

**Contact Information**

Address correspondence to Prof. Shulin Yang at Institute of Animal Sciences, Chinese Academy of Agricultural Sciences, No. 2 Yuanmingyuan West Road, Beijing 100193, P.R. China

Tel.: 86-10-62818180, Fax: 86-10-62813822, E-mail: yangshulin@caas.cn

**Supplementary information**

**Figures**

**Figure S1.** Validation of RNA-Seq data by qRT-PCR. Eight lncRNA genes in the livers of minipigs associated with lipid metabolism, chemokines, and immune response were selected for validation. Data are presented as the fold difference between the HFHSD and control groups. Each gene was normalized to glyceraldehyde-3-phosphate dehydrogenase (GAPDH).

**
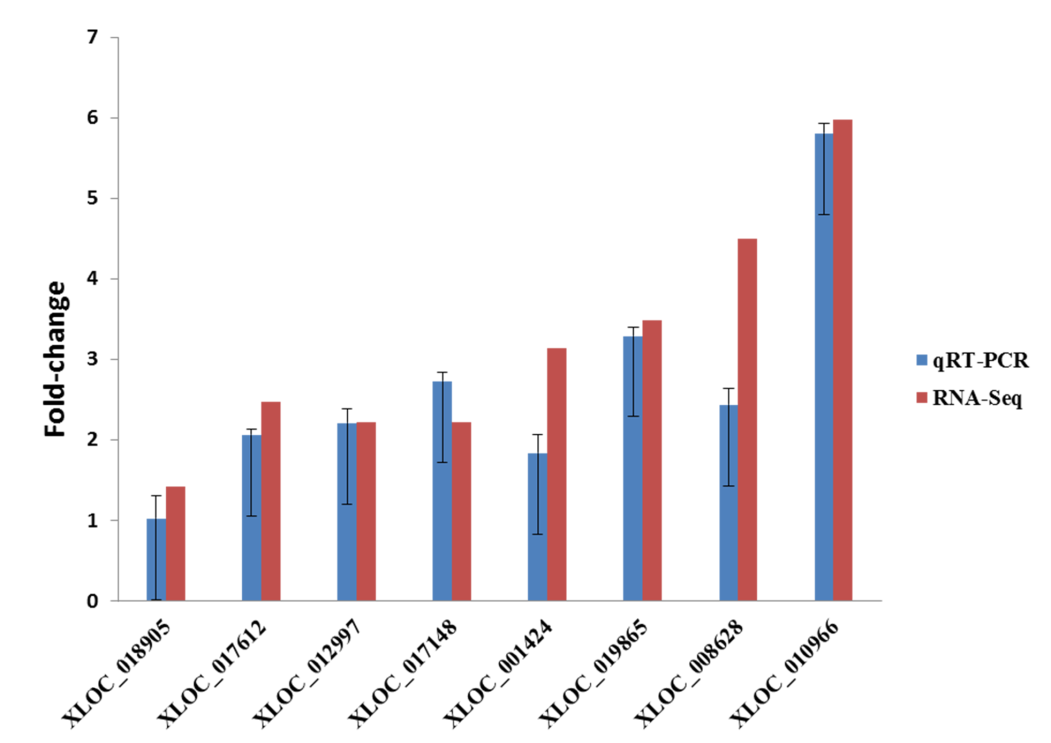
**

**Figure S2.**

**The differentially expressed mRNAs between the HFHSD and control groups; GO terms analysis and DAG figure for biological processes, molecular function, and cellular components.**


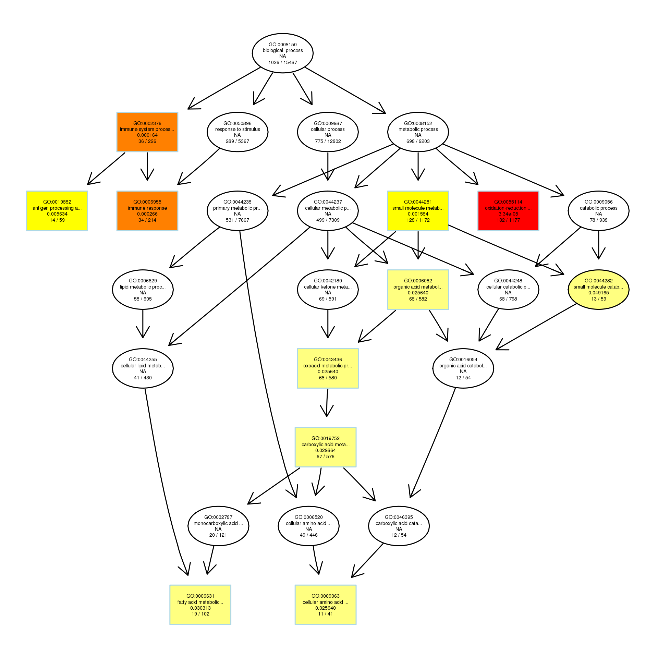

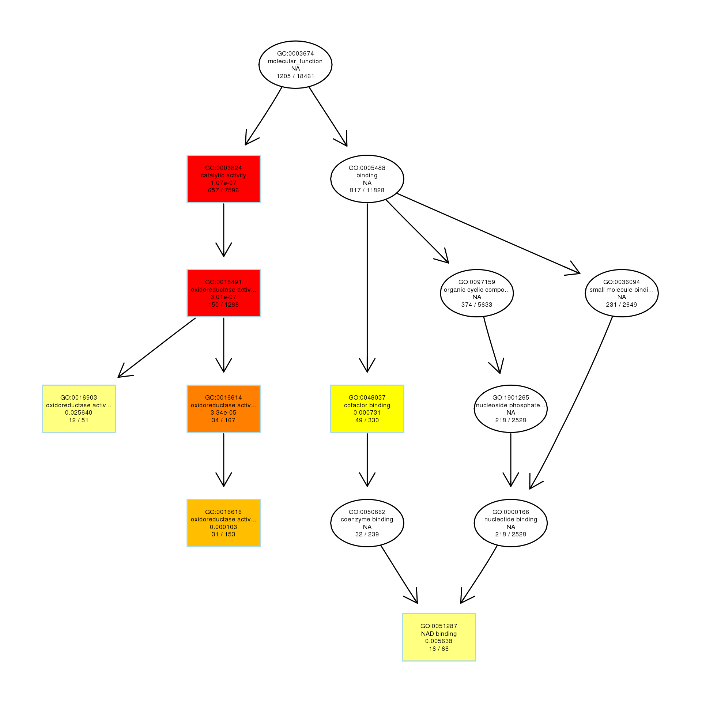

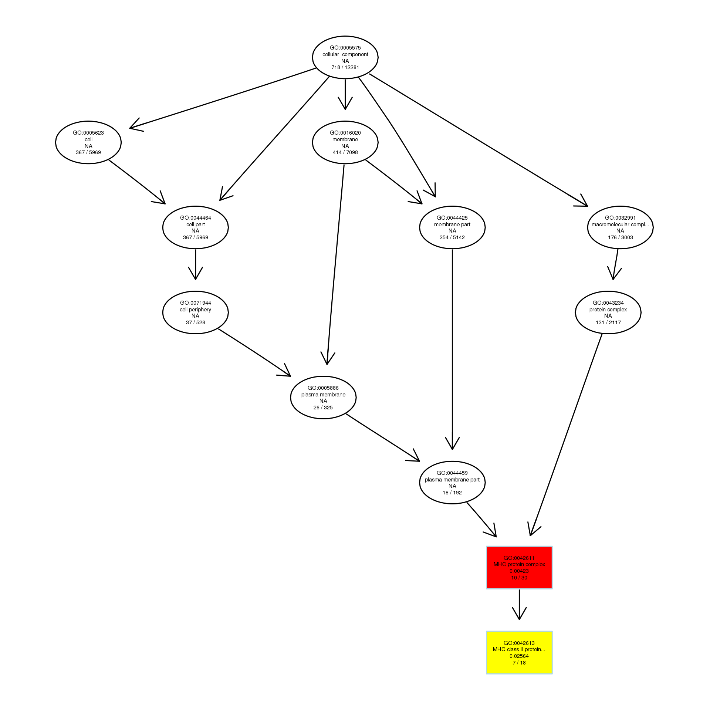


**Tables**

**Table S1 Primer pairs selected for lncRNA analysis by qRT-PCR in Bama minipigs**

**Table S2 The distribution of reads in the livers of all the minipigs.**

**Table S3 Clean reads mapped to the pig genome.**

**Table S4 The reads classified by RNA category.**

**Table S5** **The lncRNA prediction using four coding potential predicting methods.**

**Table S6** **The sequence of all the lncRNAs analyzed in the minipigs.**

**Table S7** **The mRNA statistics between HFHSD group and control group pigs**

**Table S8** **All the lncRNA statistics in the HFHSD group and control groups.**

**Table S9 The prediction of target genes of lncRNAs in cis and trans.**

**Table S10** **The target genes of the lncRNAs in *cis* that were enriched in the specific GO and KEGG pathways.**

**Table S11** **The target genes of the lncRNAs in *trans* that were enriched in the specific GO and KEGG pathways.**

**Table S1 Primer pairs selected for analysis by qRT-PCR.**

| LncRNA | Forward primer | Reverse primer |
| --- | --- | --- |
| XLOC_001424 | ATAGGGCTGTTTCCCATCATTTAC | TGGCGGCTGAGACTGACTTG |
| XLOC_008628 | ACCCATATTGGCATTGTCATCA | AGTTGTCAGTTTCTCGGAAGCAC |
| XLOC_019865 | TCTATCGCCAATATGCCACCA | CCAAAGTAGGCTATACTCCGAGCT |
| XLOC_017148 | CAATAAACACGAAACTGGCAACTC | GCACGGTGAAGCAGCAAGAA |
| XLOC_017612 | CAAACTTGGTCCACGTTTAGCC | ATCTGGGAGTCCATCCTCTGTATTA |
| XLOC_012997 | CAGGCATGAAGGGTGGAGTC | CATACCAGGAGCAAAGCAGAAAT |
| XLOC_018905 | TTAGCCGTGCGACCAAGTT | TTGTCCTCGTGGTCAAAAGTG |
| XLOC_010966 | GGACAATCCGAGGCATTTCA | CAGGTGGCAGGTTTAGTAAGCAA |

**Table S2 The distribution of reads in the livers of minipigs.**

| **Sample name** | **Raw reads** | **Clean reads** | **clean bases** | **Error rate(%)** | **Q20(%)** | **Q30(%)** | **GC content(%)** |
| --- | --- | --- | --- | --- | --- | --- | --- |
| BM_120_1 | 52542957 | 50178510 | 6.27G | 0.03 | 96.79 | 93.51 | 51.89 |
| BM_120_2 | 52542957 | 50178510 | 6.27G | 0.04 | 94.65 | 90.06 | 52.07 |
| BM_138_1 | 47453852 | 46304638 | 5.79G | 0.03 | 96.68 | 93.32 | 51.34 |
| BM_138_2 | 47453852 | 46304638 | 5.79G | 0.04 | 94.96 | 90.57 | 51.29 |
| BM_146_1 | 44080317 | 42360437 | 5.3G | 0.03 | 96.61 | 93.22 | 51.65 |
| BM_146_2 | 44080317 | 42360437 | 5.3G | 0.04 | 95.05 | 90.74 | 51.71 |
| BM_157_1 | 46786728 | 44466637 | 5.56G | 0.03 | 96.67 | 93.33 | 52.6 |
| BM_157_2 | 46786728 | 44466637 | 5.56G | 0.04 | 94.82 | 90.31 | 52.6 |
| BM_159_1 | 48986505 | 47417549 | 5.93G | 0.03 | 96.72 | 93.42 | 52.4 |
| BM_159_2 | 48986505 | 47417549 | 5.93G | 0.04 | 94.94 | 90.51 | 52.49 |
| BM_161_1 | 53076888 | 50827412 | 6.35G | 0.03 | 96.75 | 93.41 | 53.3 |
| BM_161_2 | 53076888 | 50827412 | 6.35G | 0.03 | 95.12 | 90.8 | 53.39 |

**Table S3 Clean reads mapped to the pig genome.**

| **Sample name** | **BM_120** | **BM_138** | **BM_146** | **BM_157** | **BM_159** | **BM_161** |
| --- | --- | --- | --- | --- | --- | --- |
| Total reads | 100357020 | 92609276 | 84720874 | 88933274 | 94835098 | 101654824 |
| Total mapped | 83681173 (83.38%) | 75309242 (81.32%) | 68403337 (80.74%) | 71515309 (80.41%) | 76362941 (80.52%) | 82848213 (81.5%) |
| Multiple mapped | 17771039 (17.71%) | 14059026 (15.18%) | 13635297 (16.09%) | 12977549 (14.59%) | 14435166 (15.22%) | 19598483 (19.28%) |
| Uniquely mapped | 65910134 (65.68%) | 61250216 (66.14%) | 54768040 (64.65%) | 58537760 (65.82%) | 61927775 (65.3%) | 63249730 (62.22%) |
| Read-1 | 33264653 (33.15%) | 30825192 (33.29%) | 27574875 (32.55%) | 29424326 (33.09%) | 31119014 (32.81%) | 31839305 (31.32%) |
| Read-2 | 32645481 (32.53%) | 30425024 (32.85%) | 27193165 (32.1%) | 29113434 (32.74%) | 30808761 (32.49%) | 31410425 (30.9%) |
| Reads map to '+' | 32862387 (32.75%) | 30587528 (33.03%) | 27326133 (32.25%) | 29173995 (32.8%) | 30889771 (32.57%) | 31512581 (31%) |
| Reads map to '-' | 33047747 (32.93%) | 30662688 (33.11%) | 27441907 (32.39%) | 29363765 (33.02%) | 31038004 (32.73%) | 31737149 (31.22%) |
| Non-splice reads | 42765079 (42.61%) | 44051371 (47.57%) | 39618655 (46.76%) | 42601136 (47.9%) | 42003780 (44.29%) | 39830214 (39.18%) |
| Splice reads | 23145055 (23.06%) | 17198845 (18.57%) | 15149385 (17.88%) | 15936624 (17.92%) | 19923995 (21.01%) | 23419516 (23.04%) |

**Table S4 The reads classified by RNA category.**

| **Samples** | **BM_120** | **BM_138** | **BM_146** | **BM_159** | **BM_161** | **BM_157** |
| --- | --- | --- | --- | --- | --- | --- |
| mRNA | 17625248 (66.16%) | 15072472 (59.82%) | 13334087 (57.60%) | 15484199 (59.96%) | 16460978 (62.78%) | 13882261 (56.43%) |
| misc_RNA | 462528 (1.74%) | 506257 (2.01%) | 447918 (1.93%) | 479085 (1.86%) | 422885 (1.61%) | 485711 (1.97%) |
| ncRNA | 0 (0.00%) | 0 (0.00%) | 0 (0.00%) | 0 (0.00%) | 0 (0.00%) | 0 (0.00%) |
| precursor_RNA | 187 (0.00%) | 218 (0.00%) | 162 (0.00%) | 181 (0.00%) | 213 (0.00%) | 203 (0.00%) |
| pseudogene | 17184 (0.06%) | 24862 (0.10%) | 24129 (0.10%) | 20968 (0.08%) | 14730 (0.06%) | 26636 (0.11%) |
| rRNA | 5999 (0.02%) | 8227 (0.03%) | 7839 (0.03%) | 10463 (0.04%) | 12610 (0.05%) | 12079 (0.05%) |
| tRNA | 905837 (3.40%) | 1145302 (4.55%) | 1123308 (4.85%) | 958714 (3.71%) | 990495 (3.78%) | 1089131 (4.43%) |
| Others | 7623283 (28.62%) | 8440195 (33.50%) | 8213079 (35.48%) | 8870935 (34.35%) | 8319678 (31.73%) | 9105115 (37.01%) |
